# The Utilization of Autoantibodies in Approaches to Precision Health

**DOI:** 10.3389/fimmu.2018.02682

**Published:** 2018-11-16

**Authors:** Marvin J. Fritzler, Laura Martinez-Prat, May Y. Choi, Michael Mahler

**Affiliations:** ^1^Department of Medicine, Cumming School of Medicine, University of Calgary, Calgary, AB, Canada; ^2^Inova Diagnostics Inc., San Diego, CA, United States

**Keywords:** precision medicine, precision health, biomarkers, autoantibodies, autoimmunity

## Abstract

Precision health (PH) applied to autoimmune disease will need paradigm shifts in the use and application of autoantibodies and other biomarkers. For example, autoantibodies combined with other multi-analyte “omic” profiles will form the basis of disease prediction allowing for earlier intervention linked to disease prevention strategies, as well as earlier, effective and personalized interventions for established disease. As medical intervention moves to disease prediction and a model of “intent to PREVENT,” diagnostics will include an early symptom/risk-based, as opposed to a disease-based approach. Newer diagnostic platforms that utilize emerging megatrends such as deep learning and artificial intelligence and close the gaps in autoantibody diagnostics will benefit from paradigm shifts thereby facilitating the PH agenda.

## Introduction: tenets of medicine as a framework for precision health (PH)

The three actionable tenets of clinical medicine are: (1) disease prediction and prevention; (2) early and accurate diagnosis; (3) effective and timely treatment (1, 2). The term Precision Health (PH) was deliberately chosen here to focus on the first tenet and emphasize disease prevention and healthy living or “wellness,” which refers to maintaining longer and healthier lives, a medical imperative that is shaping global research and health policy. This has been articulated as an integrated, systems approach referred to as “P4 medicine”: Predictive, Preventive, Personalized, and Participatory (3). The paradigm shift to PH is fostered by the emergence of multi-analyte diagnostic technologies, individual, real-time health information, deep learning (DL) and artificial intelligence (AI) approaches to “big data” (4–7). DL and AI are a part of our daily lives; their utility has enabled optimized travel, efficient exercise, social networking, finance management, and is beginning to permeate into healthcare. By definition, AI is comprised of supervised learning algorithms varying in complexity, which are able to profile patterns in data sets that are not immediately obvious using uni-variate approaches. The rapid growth and accessibility of AI-related technologies allows abstraction of information that may not have been immediately obvious, enabling profiling, personalizing, and delivering improved health care to each patient. Taken together, it is anticipated that these approaches to PH will translate into decreased healthcare expenditures (HCE) where the “VALUE PROPOSITION” is expressed as markedly improved clinical OUTCOMES as the numerator and COSTS as the denominator (8). Because of this “value proposition,” HCE in PH become an investment rather than a cost (9).

A successful PH paradigm is dependent on disease prediction and prevention through timely intervention (10, 11). A crucial challenge is how to shift the clinical focus to timely intervention in individuals who will progress from pre-clinical to undifferentiated autoimmune disease and then to clinically active systemic autoimmune diseases (SAIDs), the latter with attendant high morbidity and/or mortality. Keys to this approach will not only focus on healthy living and earlier, more accurate diagnosis, but curbing HCE due to decreased hospital admissions and readmissions, evidence-based decisions on expensive therapeutics, and decreased physician visits because the individual will have a more clearly defined participatory clinical care pathway (12). We touch on evidence-based and informed approaches to interventions for pre-clinical autoimmune disease that have emerged and are evolving.

## Compelling reasons for precision health

There are many compelling reasons why healthcare professionals attendant to SAIDs should embrace PH. First, it is well established that advanced disease and accompanying morbidity is often present at the time of diagnosis of SAIDs [e.g., systemic lupus erythematosus (SLE), systemic sclerosis (SSc), rheumatoid arthritis (RA), Sjögren's syndrome (SjS)]. For example, approximately 30% of SLE have kidney disease at inception or at the time they are first seen by a specialist (13) and 13% of SSc will die prematurely within 3 years of diagnosis (14). In RA, several studies have shown that all features of chronic synovial inflammation can be found within weeks to months after the first clinical evidence of arthritis, providing evidence that asymptomatic synovial inflammation may precede the development of clinical signs and symptoms in RA (15, 16). A key factor responsible for advanced morbidity and early mortality at the time of diagnosis is the time that has elapsed from the onset of symptoms to diagnosis and initiation of therapy (10, 17–19). Depending on the SAID, the interval from first symptom to diagnosis ranges from 2 to 20 years and may even be longer if the onset is in the elderly (20, 21).

Second, increased HCE on SAIDs are highly correlated with advanced disease. For example, the annual direct per patient costs for SLE was as high as $71,334.00 (2015 US dollars) in those with lupus nephritis (reviewed in (13)). A review spanning 2000–2009 found the direct costs were related to inpatient (16–50%) and outpatient (24–56%) services, followed by medications (19–30%). This is in stark contrast to HCE on a SLE patient without kidney disease where the HCE ~$5,000 USD/year (22) [reviewed in (1, 13)].

Third, clinicians will be able to make more informed therapeutic choices based on pathogenesis-based approaches that inform clinical trials (23) and individual therapies (24). The clinical misadventures and attendant HCE in “one size fits all” or “trial and error” approaches are replaced by evidence or big data based methodologies focused on the “right patient,” “right drug,” “right dose,” at the “right time” (2). Lastly, SAIDs are typically chronic conditions comprised of heterogeneous disease phenotypes. Understanding the underlying molecular mechanisms and the drivers of specific SAIDs will open new avenues for targeted treatment and improved outcomes.

## Key components of PH

To effectively implement PH, attention should be given to eight key components of the “healthcare system,” which we refer to as the “P8” (Table 1). Our proposed P8 health paradigm is based in part on the P4 medicine approach proposed by Hood and colleagues (25). In effective PH, the approach is PROACTIVE, the individual PATIENT is the focus and they must be active participants (PARTICIPATORY) in promotion of wellness in addition to monitoring and surveillance of the condition (26). A meaningful impact on PERSONAL WELLNESS and HCE will require the identification of risk factors, clinical parameters, and biomarkers that are PREDICTIVE of and then used as an approach to case finding followed by timely interventions and PREVENTION of disease. The identification of relevant and clinically meaningful biomarkers should meet criteria included in the SSSMAARTT biomarkers acronym (Sensitive, Specific, Stable, Measurable, Actionable, Added value, Realistic, Timely, and Titratable) (27).

**Table 1:** Components of Precision Health: a P8 approach.

1	PATIENT (individual) is the focus
2	PROACTIVE approach
3	PREDICTION of disease
	SSSMAARTT biomarkers^*^
	Risk factors
4	PREVENTION of disease
5	PERSONAL WELLNESS is emphasized
6	PROSPECTIVE
	Real time history, physical exam
	Real time biomarkers
7	PERSONALIZED database
8	PARTICIPATORY surveillance

* *SSSMAARTT = (Sensitive, Specific, Stable, Measurable, Actionable, Added value, Realistic, Timely, and Titratable*.

The role of healthcare providers in SAID should be PROSPECTIVE in nature by incorporating real time history, physical exam(s), biomarker results, and, where needed and available, molecular imaging (28, 29), wearable devices (30), and other investigational tools. Taken together, comprehensive individualized information is captured in PERSONALIZED electronic medical records (31, 32). The P8 approach inevitably leads to the generation of “big data.” This introduces unique computational and statistical challenges that require a new paradigm (4, 5, 7) and innovative thinking such as the use of DL and AI to reclassify disease and stratify patients based on “molecular taxonomy” or the “immunobiome” (33, 34).

Despite the promise and imperative of PH, it will likely take at least a decade before healthcare providers fully embrace and implement these concepts. One of the reasons could be the perception that the PH agenda clashes with the “less is more” movement (35, 36). However, we believe that the reticence, for the most part, is due to lack of training in PH and of clearly defined clinical care pathways focusing on prevention through timely interventions. Indeed, actionable strategies for SAID prevention have already been proposed for SLE (13), SSc (37), and RA (38–41) (Table 2). Even then, many healthcare providers are more comfortable focusing on “curing” advanced disease rather than preventing it. Hence, effective P8 PH requires extraordinary efforts to educate healthcare providers and administrators (42).

**Table 2:** Overview of prevention trials for rheumatoid arthritis.

**Trial name**	**Drug**	**Initiator/Comment**
StopRA (Strategy to prevent the onset of clinically apparent rheumatoid arthritis)	Hydroxychloroquine	University of Colorado, Denver, USA
PRAIRI (Prevention of RA by Rituximab)	Rituximab	Academic Medical Center, Amsterdam
ARIAA (Arthritis prevention in the preclinical phase of rheumatoid arthritis with Abatacept)	Abatacept	Sponsored by abatacept's manufacturer, Bristol-Myers Squibb
APIPPRA (Abatacept reversing subclinical inflammation as measured by MRI in ACPA positive Arthralgia)	Abatacept	Sponsored by abatacept's manufacturer, Bristol-Myers Squibb
TREAT EARLIER (Treat early arthralgia to reverse or limit impending exacerbation to rheumatoid arthritis)	Methylprednisolone	Leiden University Medical Center and Erasmus Medical Center in Rotterdam, both in the Netherlands.
STAPRA (Statins to Prevent Rheumatoid Arthritis)	Atorvastatin	University of Amsterdam

One of the challenges that needs to be addressed is having a proper screening or triage system for individuals at risk of developing SAIDs (43, 44). In addition, based on the risk level, a sequential and timely approach for intervention is desirable to maximize effectiveness while maintaining safety. This approach to primary prevention includes the removal or modification of lifestyle risk factors (e.g., smoking, weight loss, dietary supplement intake) or the initiation of pharmacological interventions such as timely treatment in high risk cases (e.g., with hydroxychloroquine) if prevention trials prove successful (34, 35) (secondary intervention).

## Autoantibodies and other biomarkers as predictors of disease and effective therapy

In current practice, autoantibodies are most often used to confirm the diagnosis and classify SAIDs but are also becoming increasingly important biomarkers that predict complications and/or comorbidities. Take for instance the 2012 Systemic Lupus International Collaborating Clinics classification criteria where patients must fulfill at least one clinical as well as immunologic criteria, which include antinuclear antibodies (ANA), anti-double stranded DNA (dsDNA), anti-Smith (Sm), and anti-phospholipid antibodies (45). In addition to a classification criterion, anti-dsDNA is associated with SLE nephritis (46) and can be used to monitor disease activity (47, 48). Being able to identify a patient's risk for disease complications or organ involvement based on their autoantibody profile allows clinicians to be more vigilant about screening and monitoring patients who have established disease. This has implications for the management of patients with SAIDs including SSc and myositis, where the presence of certain autoantibodies should prompt a more aggressive approach to investigation (e.g., cardiopulmonary testing, malignancy screening) and therapy (33, 49, 50).

An understanding of which autoantibodies are predictive of disease is derived from studies of patients with undifferentiated connective tissue disease (UCTD) (51). These patients generally have a milder form of a SAID that may eventually evolve into a defined SAID. In a retrospective study of 148 patients with UCTD and anti-SSA/Ro60 antibodies, ~25% developed a well-defined SAID within a short time (4.5 years) (52). Most of these patients developed SjS (50%) or SLE (30.5%). In the same study, the presence of anti-dsDNA along with anti-SSA/Ro60 antibodies was predictive of SLE development. Other small cohort studies have also shown that UCTD patients who eventually developed SLE had one of anti-dsDNA or anti-SSA/Ro60 antibodies at baseline and at diagnosis of SLE, they had developed the other antibody (53). In a more recent retrospective review of 98 UCTD patients, 14% developed into a defined SAID (54) in which the presence of an ANA titer ≥1/640 (OR 7.00 [1.99–24.66], *p* = 0.002) and anti-centromere positivity (OR 3.77 [1.03–13.79], *p* = 0.045) at baseline as well as other clinical features were associated with the development of definite SAID.

A subtype of UCTD patients with some manifestations of SLE who did not meet full classification criteria were referred to as incomplete SLE (ILE). Between 10 and 60% of patients with ILE progressed to complete SLE, usually within 5 years of disease onset (55–57). In a seminal study of 130 former military SLE patients whose sera were available from the USA Department of Defense Serum Repository, Arbuckle et al. (58) detected at least one SLE-related autoantibody before the diagnosis of complete SLE and first clinical manifestations of ~90% of subjects. ANA, anti-SSA/Ro60 and anti-SSB/La antibodies were the earliest autoantibodies to appear, while others such as anti-Sm and anti-U1RNP antibodies appeared only months prior to diagnosis. The mean interval from earliest autoantibodies detected to diagnosis of complete SLE ranged from 3.7 years for anti-SSA/Ro60 to 0.9 years for anti-U1RNP. Other studies have shown that anti-cardiolipin (aCL) antibodies are significant predictors of complete SLE development (56, 59). McCain et al. demonstrated that 24/130 (18.5%) SLE patients were positive for IgG and/or IgM aCL prior to SLE diagnosis (59). The antibodies appeared as early as 7.6 years prior to SLE diagnosis with a mean onset occurring 3.0 years before SLE diagnosis. The presence of aCL also seemed to predict a more severe clinical outcome including more frequent renal disease, central nervous system disease, thrombocytopenia, and clotting events.

Patients with features of SSc but not meeting classification criteria have been referred to as very early systemic sclerosis (60–62). Once again, the idea of an earlier diagnosis of this disabling disease is to allow earlier interventions designed to block or slow progression to severe morbidity. The current diagnostic and classification criteria limit the ability to detect early disease because they typically depend on features that are the sequelae of the disease. In one study of 60 early SSc patients, the presence of autoantibodies (anti-Scl-70, centromere, and/or anti-RNA polymerase) was the most important predictor of faster progression to SSc, particularly in those with preclinical internal organ (heart or lung) involvement at baseline (63).

Using RA as another example, < 50% of anti-citrullinated peptide antibodies (ACPA) positive individuals will develop RA within 3 years of follow-up (64). The risk of developing RA therefore depends on many factors that can be divided into three categories: the modifiable (e.g., behavioral: smoking, dietary, environmental), fluctuating or progressive (e.g. autoantibodies and other biomarkers), and the constant (e.g. germline genetics) (65–69). The pre-clinical phase of RA and other SAIDs may be mitigated by modification of the risk profile (70). It has been established that the presence of three biomarkers (ACPA, rheumatoid factor, and anti-carbamylated protein antibodies) are highly discriminatory in correctly identifying RA in a cohort of early arthritis (71–73). When comparing the presence of one, two or three autoantibodies to the patients with zero autoantibodies, the odds ratio (OR) of having the diagnosis RA significantly increased from 3.8 (95 % CI 2.9–5.0) for the patients with one autoantibody, to 20.9 (95 % CI 12.7–34.3) for the group with two and finally to 112.2 (95 % CI 52.4–240.5) for the individuals with three autoantibodies (74). It should be emphasized that additional studies need to determine the predictive value of autoantibody profiles in prospective, longitudinal cohorts of individuals with/without early clinical features of RA.

A rapidly emerging area of evidence indicates that the glycosylation of antibodies can also provide added value in prediction of RA. It has been known for more than two decades that glycosylation of antibodies is associated with autoimmunity (75, 76). Several studies have demonstrated that the Fc portion of IgG shows different glycosylation patterns in patients with autoimmune diseases when compared to healthy individuals [reviewed in (76)]. In healthy individuals, the glycosylation site is typically fully glycosylated IgG. In addition, there is growing evidence that the differences in glycosylation also reflect variations in disease activity. Lastly, the ratio between G0/G1 glycosylation type also changes during the conversion from pre-clinical to clinical phase. In contrast to the Fc de-glycosylation, the Fab regions of antibodies exhibit increased levels of glycosylation in RA compared to healthy individuals which alters properties of the antibody in a variety of ways. Importantly, the glycosylation occurs as part of the somatic hypermutation of the complementary-determining regions and might have impact on the antigen binding characteristics. In a recent study, it was shown that the affinity of ACPA can either increase or decrease based on the glycosylation of their variable domains (77, 78). In addition, the stability of the antibody can increase after Fab glycosylation which also can lead to the formation of immune complexes. Therefore, the glycome status of an individual might provide valuable insights in the prediction of SAIDs (79, 80). Furthermore, this might even pave the way for new treatment approaches through glycoengineering of antibodies (81, 82).

In addition to autoantibodies, other biomarkers included in omics technologies (e.g., genomics, ribonomics, proteomics, glycomics, metabolomics, etc.) are also becoming key components of PH (83, 84). An important contribution of these multi-analyte technologies is to help understand the pathogenic processes throughout the entire course of the disease. This understanding will be key to guide patient stratification for clinical trials and evidence-based interventions as opposed to the “trial and error” approaches that are prevalent today (23). In this context, advances in molecular medicine are increasingly based on the use of biomarkers as drug development tools (85). Companion diagnostics are expected to identify individuals with a higher chance to benefit from a particular treatment and in this way, biomarkers can increase the success rate of drug development programs and accelerate the availability of new therapeutics (86, 87).

## Filling knowledge and technological gaps

To make progress in the use of autoantibodies in the PH agenda, knowledge and technological gaps must be addressed. First, the parameters and mechanisms that chart or trigger the evolution of very early or undifferentiated SAIDs to full clinical disease must be clarified (88). Examples have been discussed above but longitudinal studies of larger early SAID and UCTD cohorts, or even apparently healthy cohorts using newer multi-analyte technologies are required to more accurately classify and predict disease.

In addition to clinical gaps, there are technological gaps that also need to be addressed. With respect to autoantibody testing, universally accepted, standardized, and cost-effective follow-up testing algorithms need to be developed. Although the ANA indirect immunofluorescence (IIF) test is regarded as the screening test of choice for some SAIDs, recent evidence suggests that follow-up testing algorithms might be inverted wherein multi-analyte solid phase assays (MSPA) (Table 3) that detect antibodies to disease-specific autoantibody targets is a more cost-effective approach (89–91). Samples that have negative multi-analyte array test results might then be tested by ANA IIF to determine if antibodies to targets not included in the MSPA are detected. Although older thinking indicated that only a single autoantibody was required to confirm a diagnosis, this can lead to a diagnostic dilemma because many sera from SAID patients typically have multiple autoantibodies. Furthermore, each antibody could have added value not only for accurate diagnosis but also for disease stratification and on evidence-based approaches to therapy and prognosis. The sensitivity of many autoantibody assays today, including MSPAs, is limited to well-characterized targets although it is known that the B-cell repertoire in a given SAID is often much more complex. Hence, a significant proportion of SAIDs are potentially incorrectly labeled “sero-negative” (92). It is anticipated that the sensitivity, specificity and predictive value of autoantibody testing can be increased by including AI approaches to MSPA, a paradigm shift referred to as multi-analyte arrays with algorithmic analyses (MAAAA) (93).

**Table 3:** Current and emerging autoantibody detection technologies.

• Indirect immunofluorescence (IIF)
• Cell based assays
• Enzyme-linked immunoassays (ELISA)
• Multi-analyte solid phase assays (MSPA)
° Antigen arrays on planar surfaces: line immunoassays, dot blots
° Addressable laser bead assays (ALBIA)
° Particle-based multi-analyte technologies (PMAT)
° Chemiluminescence (CIA)
° Multiplexed point of care diagnostics: *“Lab on a chip”*
° Multi-analyte arrays with algorithmic analysis (MAAAA)
• Mass spectroscopy
• Electrochemiluminescence arrays
• Nanotechnology—nanobarcodes

In summary, PH applied to SAID is anticipated to create paradigm shifts in the use and application of autoantibodies. Autoantibodies combined with other multi-analyte “omic” profiles will form the basis of disease prediction, and subsequently, earlier intervention linked to disease prevention strategies. As medical intervention moves to disease prediction and intent-to-prevent, diagnostics will include an early symptom/risk-based approach, as opposed to a disease-based approach. Newer platforms and technologies adaptable to the paradigm shifts that help close the gaps in autoantibody profiling will facilitate the PH agenda. In this setting, DL and AI will be key to interpretation of “big data” from multi-analyte autoantibody arrays and other biomarker testing. The current status and future of PH in RA and SLE has recently been discussed at the Precision Medicine in Autoimmunity (PMA) meeting (http://www.precisionmedicineautoimmunity.org/) which will likely provide the basis for new collaborative work towards a more precise approach to medicine in autoimmune conditions.

## Author contributions

MF: Conceived topic, wrote outline, and parts of manuscript and primary edits. LM-P, MM, and MC: Literature review, wrote parts of and edited the manuscript.

### Conflict of interest statement

MF is a consultant to Inova Diagnostics, (San Diego, CA, USA) and Werfen International (Barcelona, Spain). MM and LM-P are employees of Inova Diagnostics, a company that manufactures and sells autoantibody assays. The remaining author declares that the research was conducted in the absence of any commercial or financial relationships that could be construed as a potential conflict of interest.

## Abbreviations

aCL: anti-cardiolipin
ACPA: anti-citrullinated peptide antibodies
AI: artificial intelligence
ANA: anti-nuclear antibody
DL: deep learning
dsDNA: double-stranded DNA
HCE: healthcare expenditures
IIF: indirect immunofluorescence
MSPA: multi-analyte solid phase assays
PH: precision health
RA: rheumatoid arthritis
SAID: systemic autoimmune disease
SjS: Sjögren's syndrome
SLE: systemic lupus erythematosus
Sm: smith
SSc: systemic sclerosis
UCTD: undifferentiated connective tissue disease.
